# P-1077. Colistin Use and the Emergence of Pathogens Intrinsically Resistant to Colistin in Critically-ill Patients: a Hidden Consequence?

**DOI:** 10.1093/ofid/ofaf695.1272

**Published:** 2026-01-11

**Authors:** Sruthi Menon, Murali Alagesan, Nawaf Abdulla, Aruloli Mohambourame, Santhosh Raja, Krishna S Nair

**Affiliations:** PSG IMSR and Hospitals, Coimbatore, Tamil Nadu, India; PSG Institute of Medical Sciences and Research, Coimbatore, Tamil Nadu, India; PSG Institute of Medical Sciences and Research, Coimbatore, Tamil Nadu, India; PSG Institute of Medical Sciences and Research, Coimbatore, Tamil Nadu, India; PSG Institute of Medical Sciences and Research, Coimbatore, Tamil Nadu, India; PSG Institute of Medical Sciences and Research, Coimbatore, Tamil Nadu, India

## Abstract

**Background:**

Inadequate infection control and irrational antibiotic use contribute to the rise of multi-drug resistant (MDR) organisms. Use of colistin for treating MDR infections leads to the selection of bacteria that are intrinsically resistant to colistin. However there is a scarcity of studies assessing the incidence of isolates intrinsically resistant to colistin in critically-ill patients. This study aimed to determine the prevalence and factors associated with gram-negative rods intrinsically resistant to colistin in critically ill patients.

**Figure:**
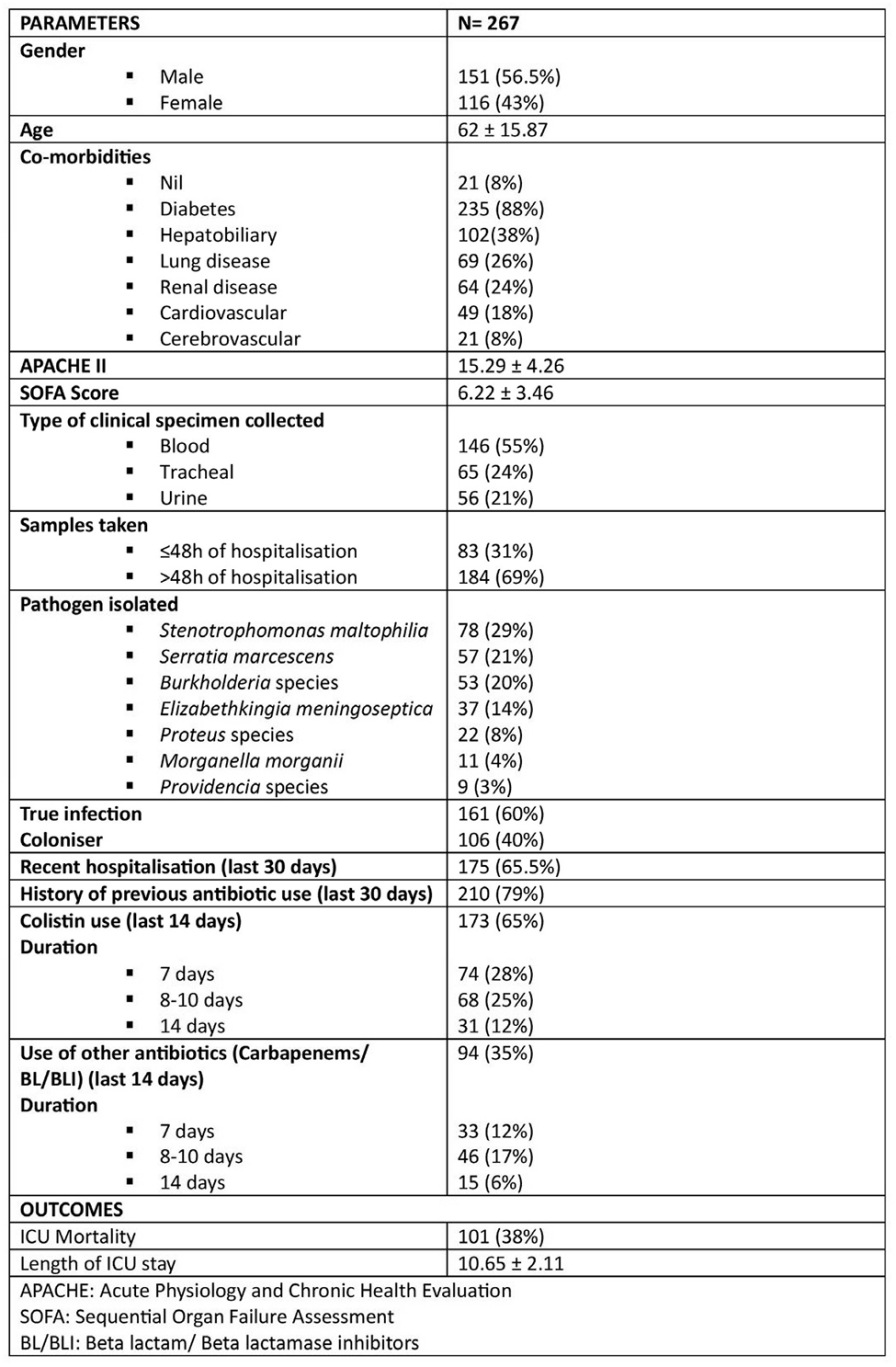
Demographic data and Outcomes

**Figure:**
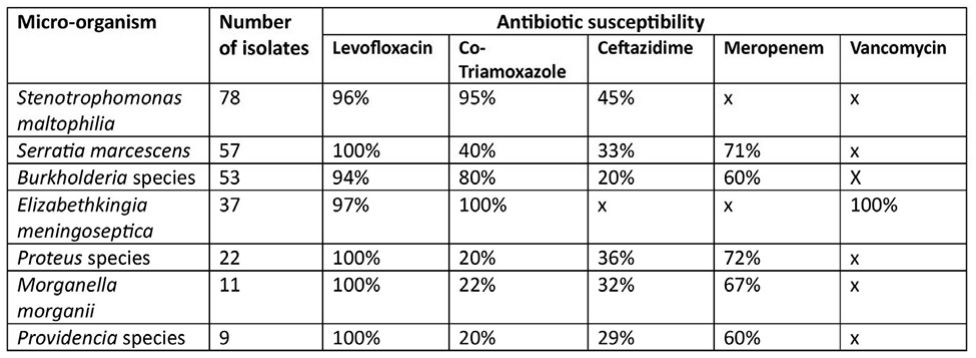
Antibiotic susceptibility

**Methods:**

We retrospectively evaluated critically-ill patients at a tertiary care hospital in India from June 2022 to June 2024. Inclusion criteria included isolation of gram-negative rods that are intrinsically resistant to colistin from clinical specimens : blood, tracheal and urine. Clinical and demographic data were analysed using SPSS version 28. A p-value < 0.05 was considered significant.

**Figure:**
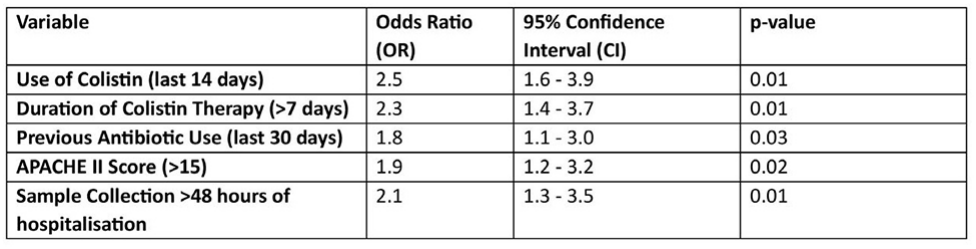
Multivariant analysis using logistic regression

**Figure:**
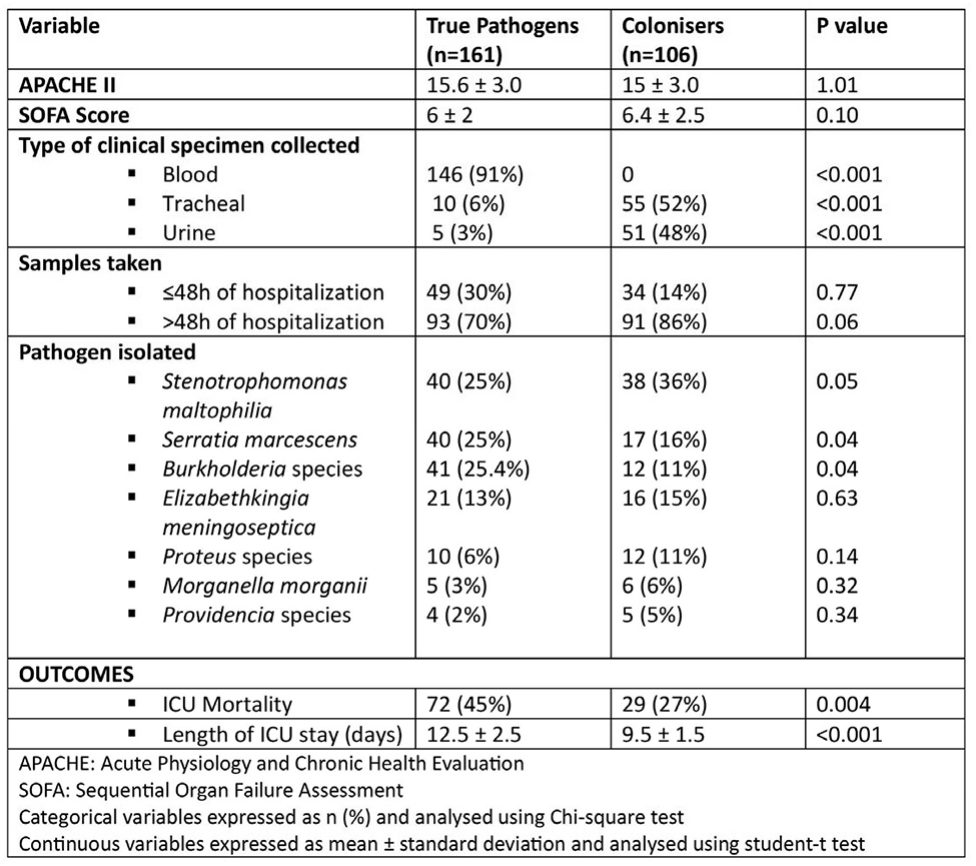
True pathogens vs colonisers

**Results:**

A total of 267 isolates were included: *Stenotrophomonas maltophilia* (29%), *Serratia marcescens* (21%), *Burkholderia* species (20%), *Elizabethkingia meningoseptica* (14%), *Proteus* species (8%), *Morganella morganii* (4%) and *Providencia* species (3%). 65% of patients had prior exposure to colistin, 79% had previous antibiotic exposure and 65.5% had been hospitalised within the last 30 days. Of the 267 isolates 60% were true pathogens and 40% were colonisers. ICU stay and mortality significantly increased with true pathogen presence.

Multivariate logistic analysis identified colistin use >7 days, prior antibiotic exposure in the last 30 days, a high APACHE II score and clinical sample collection >48 hours post hospitalisation as predictors for isolating colistin-resistant gram-negative rods.

**Conclusion:**

Our findings highlight the link between colistin use and the emergence of intrinsically resistant pathogens. Judicious colistin use, antimicrobial stewardship and strict infection control are essential. In cases of new-onset sepsis during colistin therapy it is essential to consider coverage for pathogens that are intrinsically resistant to ensure appropriate empirical therapy and avoid treatment delays. However it is also important to establish the clinical relevance of these organisms before treating them as true pathogens.

**Disclosures:**

All Authors: No reported disclosures

